# Interplay between Synaptonemal Complex, Homologous Recombination, and Centromeres during Mammalian Meiosis

**DOI:** 10.1371/journal.pgen.1002790

**Published:** 2012-06-28

**Authors:** Huanyu Qiao, Jefferson K. Chen, April Reynolds, Christer Höög, Michael Paddy, Neil Hunter

**Affiliations:** 1 Howard Hughes Medical Institute and Departments of Microbiology, Molecular and Cellular Biology, and Cell Biology and Human Anatomy, University of California Davis, Davis, California, United States of America; 2 Department of Cell and Molecular Biology, Karolinska Institutet, Stockholm, Sweden; 3 Microscopy and Imaging Facility, Department of Molecular and Cellular Biology, University of California Davis, Davis, California, United States of America; Stowers Institute for Medical Research, United States of America

## Abstract

The intimate synapsis of homologous chromosome pairs (homologs) by synaptonemal complexes (SCs) is an essential feature of meiosis. In many organisms, synapsis and homologous recombination are interdependent: recombination promotes SC formation and SCs are required for crossing-over. Moreover, several studies indicate that initiation of SC assembly occurs at sites where crossovers will subsequently form. However, recent analyses in budding yeast and fruit fly imply a special role for centromeres in the initiation of SC formation. In addition, in budding yeast, persistent SC–dependent centromere-association facilitates the disjunction of chromosomes that have failed to become connected by crossovers. Here, we examine the interplay between SCs, recombination, and centromeres in a mammal. In mouse spermatocytes, centromeres do not serve as SC initiation sites and are invariably the last regions to synapse. However, centromeres are refractory to de-synapsis during diplonema and remain associated by short SC fragments. Since SC–dependent centromere association is lost before diakinesis, a direct role in homolog segregation seems unlikely. However, post–SC disassembly, we find evidence of inter-centromeric connections that could play a more direct role in promoting homolog biorientation and disjunction. A second class of persistent SC fragments is shown to be crossover-dependent. Super-resolution structured-illumination microscopy (SIM) reveals that these structures initially connect separate homolog axes and progressively diminish as chiasmata form. Thus, DNA crossing-over (which occurs during pachynema) and axis remodeling appear to be temporally distinct aspects of chiasma formation. SIM analysis of the synapsis and crossover-defective mutant *Sycp1^−/−^* implies that SCs prevent unregulated fusion of homolog axes. We propose that SC fragments retained during diplonema stabilize nascent bivalents and help orchestrate local chromosome reorganization that promotes centromere and chiasma function.

## Author Summary

Gamete cells, such as sperm and eggs, form via the specialized cell division called meiosis. Essential and interdependent features of meiosis include the pairing, recombination, and segregation of maternal and paternal chromosomes. Chromosome pairing culminates with formation of synaptonemal complexes (SCs), zipper-like structures that connect the structural cores or axes of homologous chromosomes. Although SC is known to be important for crossover recombination, details of its function remain enigmatic. In this study, we analyze mouse spermatocytes to investigate the interplay between SC, recombination, and centromeres (the structures that direct chromosome segregation). We show that SC prevents unregulated interactions between chromosome axes. This function appears to be especially important at chromosome ends and at crossover sites where DNA exchange must be coordinated with structural exchange of chromosome axes. We also show that centromeres remain associated by short fragments of SC after general chromosome desynapsis has occurred. Furthermore, we detect a distinct type of inter-centromeric connection that persists even after centromeres desynapse. Such connections may facilitate the segregation of chromosomes that have failed to crossover. Together, our data provide new insights into the functions of SC and raise the possibility of a back-up chromosome segregation system in mammals analogous to those described in fruit flies and budding yeast.

## Introduction

The formation of gametes typically involves halving of the cellular chromosome complement from diploid to haploid. This is achieved via two consecutive rounds of chromosome segregation during the process of meiosis [1]. Prior to the first meiotic division, replicated chromosomes associate into homologous pairs and become connected along their lengths by synaptonemal complexes (SCs) [2], [3]. SCs are proteinaceous structures with a zipper-like morphology [4]–[8]. The tripartite SC structure comprises two lateral elements, inferred to be elaborations of cohesin-based homolog axes, and a central element consisting of transverse filaments that interconnect the two lateral elements [7]–[10]. SC components show tendencies for self-assembly into ordered arrays and SC formation is believed to occur via polymerization from specific nucleation sites where the homolog axes have been brought into close proximity [6], [11].

In many organisms, including plants, fungi and mammals, the template-dependent DNA-repair process called homologous recombination is coopted during meiosis to facilitate homolog pairing and synapsis [12]. In these cases, SC formation often nucleates at points where recombination brings the homolog axes together [11]. However, organisms such as *Drosophila* and *C. elegans* do not require recombination for homolog pairing and SC formation and instead have evolved dedicated chromosome pairing sites [3], [13].

In addition to promoting chromosome pairing and synapsis, recombination plays a critical function in directing the disjunction of homologs at the first meiotic division. Specifically, crossover recombination in conjunction with sister-chromatid cohesion results in structures called chiasmata that tether homolog pairs and thereby facilitate their stable biorientation on the spindle [14]–[16]. The interdependence of recombination and SCs is further highlighted by the fact that synapsis promotes crossing-over, at least in part by recruiting crossover-specific recombination factors [17], [18]. Furthermore, studies in a number of organisms imply a functional relationship between SC nucleation sites and crossovers (reviewed in [11]). Specifically, SC formation often initiates at sites where crossovers will subsequently form.

Recent studies in *Saccharomyces cerevisiae* and *Drosophila* suggest that centromeres play special roles in meiotic chromosome pairing and the initiation of SC formation [19], [20]. During early prophase in *S. cerevisiae*, centromeres undergo homology-independent “coupling”, which depends on the SC central element component, Zip1 [21], [22]. Centromere coupling is proposed to be a driving force for two-by-two chromosome association that facilitates recombination-dependent homolog pairing [22], [23]. However, analysis of recombination patterns in the *zip1* mutant support an alternative proposal that coupling helps to suppress centromere-proximal crossing-over, which is associated with chromosome nondisjunction [24] [25]–[27]. Following initial coupling, centromeres appear to act as nucleation sites for SC polymerization, although it is clear that recombination sites within the chromosome arms are also utilized [11], [28]. Consistent with a role for centromeres in nucleating SC formation, in a mutant situation where SCs are assembled independently of recombination, centromeres appear to be the exclusive sites of initiation [29]. This same study indicated that Zip3, a putative E3-ligase for conjugation of the small protein modifier SUMO, negatively regulates SC initiation between centromeres. A role for centromeres in SC formation is further supported by recent studies in *Drosophila* females, which showed that centromeres undergo SC-dependent clustering and function as SC initiation sites [19], [30], [31].

Although crossing-over is a highly efficient process, achiasmate homologs do occasionally arise. In budding yeast, Zip1-mediated centromere coupling plays an additional late role to facilitate the disjunction of achiasmate chromosomes [21], [32]. In conjunction with the spindle assembly checkpoint, this process promotes the accurate disjunction of a single pair of achiasmate chromosomes in about 90% of meioses (random segregation predicts disjunction in only 50% of cells) [32], [33]. Efficient achiasmate segregation is also observed in *Drosophila*, although a role for SC components has not been demonstrated [34].

In some mammals, SC components are also inferred to promote the disjunction of achiasmate chromosomes, specifically the sex chromosomes [35]–[37]. Typically, mammalian X and Y chromosomes synapse at short regions of homology, termed pseudoautosomal regions, where crossing-over occurs to form X-Y chiasmata [38]–[40]. However, in the Elegant Fat-tailed Mouse Opossum (a marsupial) and the Mongolian gerbil (a eutherian mammal), X-Y chiasmata are not formed, but persistent structures composed of SC proteins appear to tether the X and Y to facilitate their disjunction during anaphase [35]–[37]. Finally, in some insects, SCs are retained until anaphase I and appear to completely supersede the function of chiasmata in directing disjunction [6].

In this study we analyze the interplay between SCs, recombination and centromeres in the mouse. Immunocytological analysis of prophase spermatocytes from wild-type and mutant mice indicates that centromeres do not undergo early-stage coupling and SC assembly never initiates from centromeres. However, centromeres remain associated throughout much of the diplotene stage, connected by short SC fragments. While this general, SC-dependent centromere association appears to be lost prior to diakinesis, we detect a distinct class of inter-centromeric bridges at this stage. These structures could play a more direct role in biorienting homologs on the spindle and raise the possibility of an achiasmate segregation system in mouse.

A second distinct class of retained SC fragment is also observed during diplotene and shown to be crossover-dependent. Structured illumination microscopy reveals that these structures mark sites of developing chiasmata and are lost as the homolog axes fuse. Analysis of the SC-defective *Sycp1* mutant suggests a novel role for the SC central element in preventing inappropriate interactions between homolog axes. We discuss the idea that SC fragments retained during diplonema function to locally stabilize homolog associations and coordinate important morphological and compositional changes in preparation for chromosome segregation.

## Results

**Figure 1:**
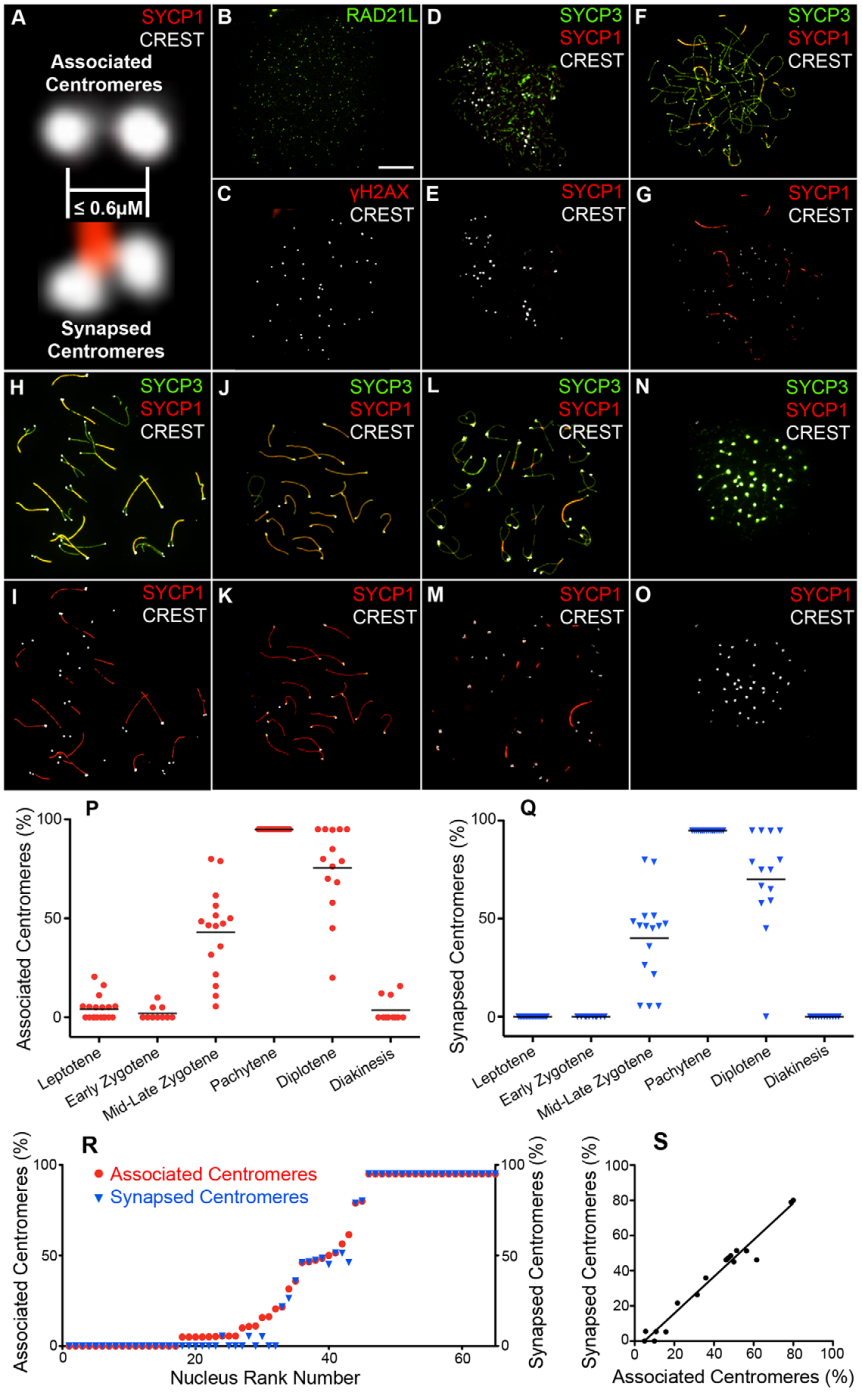
Centromere association and synapsis during meiotic prophase I. (A) Criteria for assigning “associated” and “synapsed” centromeres. Magnified images from nuclei stained with CREST (white) and SYCP3 (red) are shown. (B and C) Surface spread pre-leptotene spermatocyte nucleus immunolabeled for RAD21L (green), γH2AX (red), and CREST (white). (D–O) Representative prophase spermatocyte nuclei immunolabled for SYCP3 (green), SYCP1 (red), and CREST (white). To highlight the association status of centromeres, CREST and SYCP1 channels are shown separately. Nuclei stages are as follows: (D and E) leptonema; (F and G) early zygonema; (H and I) late zygonema; (J and K) pachynema; (L and M) diplonema; (N and O) diakinesis/metaphase. (P and Q) Levels of centromere association and centromere synapsis in individual nuclei at the various prophase stages (note that “associated centromeres” includes “synaspsed centromeres”). *P* values for comparisons of associated and synapsed groups (Mann-Whitney tests) for the various stages are as follows: leptotene, 0.0009; early zygotene, 0.078, mid/late zygotene, 0.52; pachytene, 0.99; diplotene, 0.52. (R and S) Correlations between centromere association and centromere synapsis. For the graph in (R), individual nuclei were ranked according to their level of centromere association. The graph in (S) shows the correlation for zygotene nuclei (R-squared = 0.97). The X intercept of 4.9% confirms the existence of a low level of associated, but not synapsed centromeres. Scale bar = 10 µm.

The formation and disassembly of SCs, and the pairing status of centromeres were assessed throughout meiotic prophase using immunofluorescence staining of surface-spread chromosomes from mouse spermatocytes (Figure 1; Materials and Methods). Centromeres were detected using CREST antiserum, which recognizes the constitutively centromere-associated proteins, CENP-A, -B, and -C [41]. We stringently defined “associated” centromeres as pairs of CREST foci that were ≤0.6 µm apart (regardless of whether or not they were associated with SC; Figure 1A). A cutoff of 0.6 µm was chosen because this was the maximum distance measured between synapsed CREST foci in pachytene nuclei. We also quantified frequencies of “synapsed” centromeres, which were defined as associated CREST signals that also colocalized with SC central-element protein, SYCP1 (the mammalian ortholog of budding yeast Zip1 [42]; Figure 1A).

To identify nuclei in very early prophase (pre-leptonema), slides were co-stained for the cohesin marker, RAD21L (a meiosis-specific kleisin [43]–[45]) and the DSB marker, γH2AX (a histone H2A variant that is rapidly phosphorylated at sites of DSB formation [46]). Pre-leptonema nuclei were defined as being RAD21L positive and γH2AX negative (Figure 1B and 1C). Other prophase stages were defined using standard cytological criteria by immunostaining for the homolog axis component, SYCP3, and SC central element protein, SYCP1. During leptonema, short stretches of SYCP3 staining mark the developing homolog axes (Figure 1D and 1E). SYCP3 axes elaborate into contiguous structures throughout zygonema and homologs progressively synapse (Figure 1F–1I). Cells reach pachynema when all the autosomes are fully synapsed, as shown by contiguous SYCP1 staining (Figure 1J and 1K). Progressive desynapsis of homologs occurs during diplonema to reveal homolog axes connected by nascent chiasmata (Figure 1L and 1M). Subsequently SYCP3 axes breakdown and cells enter diakinesis (Figure 1N and 1O). Diplotene and diakinesis stages are also marked by a pronounced enrichment of SYCP3 staining at the centromeric ends of the telocentric mouse chromosomes [10], [47].

### Centromere Association during Meiotic Prophase

Figure 1 shows representative nuclei from each stage of meiotic prophase. Quantification of centromere association and centromere synapsis throughout these stages is presented in panels 1P and 1Q, respectively. This analysis reveals several key features of prophase centromere behavior in mouse. Analogous observations are made by Bisig et al. in the accompanying study [48].

#### Centromeres are not coupled prior to zygonema

In pre-leptotene and leptotene stage nuclei, low levels of centromere association are observed (<21%), but centromeres are never associated with SYCP1 (Figure 1B–1E). In fact, unlike budding yeast Zip1, no chromosome-associated SYCP1 staining is detected in spread nuclei at these stages. Thus, a metastable pre-DSB centromere-association process, analogous to the Zip1-dependent centromere-coupling described in budding yeast, does not occur in mouse.

#### Synapsis does not initiate at centromeres

During early zygonema, initial stretches of SC detected by SYCP1 staining are not associated with centromeres (0 out of 92 SYCP1 stretches; 10 nuclei analyzed), which is reflected in the very low levels of centromere association and the absence of synapsed centromeres in these nuclei (Figure 1F, 1G and 1Q), i.e. SC formation does not initiate at centromere regions. This again contrasts budding yeast in which synapsis frequently initiates at or close to centromeres [28].

#### Centromeres are the last regions to synapse

Analysis of late-zygonema nuclei reveals that centromeres are generally the last chromosomal regions to synapse (Figure 1H and 1I). The fact that mouse chromosomes are telocentric raises the possibility that late synapsis of centromeres is a consequence of their sub-terminal location. However, we observed that the non-centromeric ends of homologs nearly always synapsed before the centromeric ends did. In 89.2% of homologs with synapsis at only one end, the synapsed end was the non-centromeric end (107/120 chromosomes from 16 nuclei). Therefore, late synapsis appears to be an inherent feature of centromeres and is not a consequence of their terminal location in mouse. Consistent with this inference, late synapsis of centromeres has been noted in a number of organisms with metacentric chromosomes [11], [49]–[52], including humans [53].

#### Centromere association correlates with centromere synapsis

During zygonema, levels of centromere association and centromere synapsis are closely correlated (Figure 1P, 1Q, 1R and 1S). Overall, 90.4% of associated centromere pairs detected in zygotene nuclei are also synapsed (123/136, 19 nuclei). However, low levels of associated but not synapsed centromeres are detected. These ostensibly synapsis-independent centromere associations could be *bona fide* interactions, but may also result from a low level of fortuitous colocalizations, or be driven by closely adjacent synapsis that hasn't yet converged on the CREST signals. Overall, these data imply that the close juxtaposition of homologous centromeres during mouse meiosis is driven primarily by advancing synapsis that initiated at a distal site.

#### The centromeres of sex chromosomes do not pair in pachynema

Stable pairing and synapsis of mouse sex chromosomes occurs between the pseudoautosomal regions, short (≤1 Mb) stretches of homology located at the non-centromeric ends of the X and Y [54]. Given the observations above, that SC formation does not initiate at centromeres and centromere association appears to be dependent on synapsis (that must have initiated from an adjacent site), we predicted that the sex chromosome centromeres should never be paired. Indeed, this is the case: in 20 pachynema nuclei, in which PAR synapsis was observed, the centromeres of the X and Y chromosomes were never associated.

#### Centromeres and nascent chiasmata are the last sites to desynapse

As previously described [6], [49], [50], [52], centromeres and chiasma sites behave distinctly from other chromosomal regions as homologs desynapse during diplonema (Figure 1L and 1M). First, centromeres are typically some of the last regions to separate, with high levels of association remaining until the end of diplonema (Figure 1P, 1Q). Second, the last remnants of SYCP1 staining are invariably localized with associated centromeres as well as with nascent chiasmata (discussed below). Notably, even for bivalents in which desynapsis appears to have initiated adjacent to the centromeres, a focus of SYCP1 often remains colocalized with the paired CREST signals. Thus, centromeres and crossover sites are relatively refractory to desynapsis.

#### Centromere association is lost by late diplonema

As the level of homolog desynapsis reaches ≥80%, centromere association is lost. By diakinesis, as axial SYCP3 structures diminish and centromere-associated SYCP3 further accumulates, homolog centromeres are infrequently associated and SYCP1 is undetectable (Figure 1N, 1O, 1P and 1Q).

### Synapsis Does Not Initiate at Centromeres in the Absence of Recombination or the Mammalian Zip3 Ortholog, Rnf212

In budding yeast *spo11* mutants, which fail to initiate recombination, persistent Zip1-mediated centromere coupling is observed [22]. Moreover, in the absence of recombination, centromeres become the primary sites of SC initiation in budding yeast [29]. Therefore, we analyzed the relationship between centromeres and SC in *Spo11^−/−^* knock-out mice (Figure S1). Neither early centromere-associations nor preferential initiation of SC formation from centromeres were observed in *Spo11^−/−^* spermatocytes.

Budding yeast Zip3 is a RING-domain protein and putative E3-ligase for the ubiquitin-like modifier, SUMO [55], [56] (also see [57]). While yeast *zip3* mutants show a general synapsis defect, the vast majority of SCs that do form are initiated between centromeres, leading to the proposal that Zip3 specifically inhibits SC initiation between centromeres [28], [29]. We recently constructed a knock-out mutation of the mouse Zip3 homolog, *Rnf212* (A.R., H.Q., J.K.C. and N.H., unpublished data), allowing us to test the idea that RNF212 has an analogous inhibitory function in mammals [58]. Analysis of initial SC stretches in zygotene-stage *Rnf212^−/−^* spermatocytes shows that, as in wild type, SC formation does not initiate between centromeres (Figure S2). Thus, absence of the Zip3 homolog, RNF212, does not permit SC to initiate between centromeres in mammals.

### SC Remnants during Diplonema Contain SC Central Element Components, SYCE1, SYCE2, SYCP1, and TEX12

**Figure 2:**
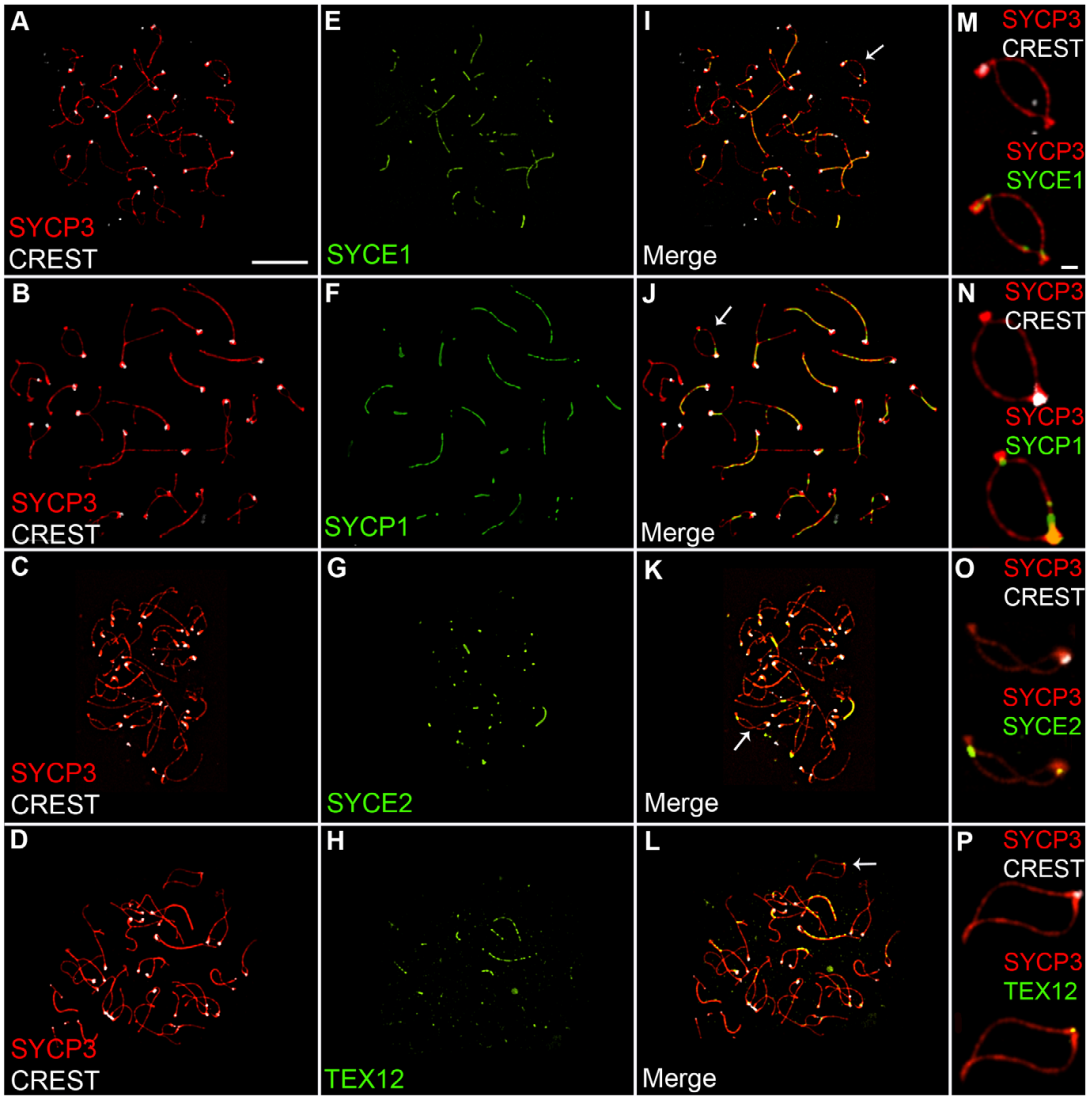
Localization of synaptonemal complex central element components during diplonema. Diplotene stage spermatocyte nuclei, immunolabeled for SYCP3 (red), CREST (white) and various SC central-element proteins (green). (M–P) Magnifications of bivalent chromosomes (indicated by arrows in panels I–L), highlighting the localization of SC central element proteins to centromeres and nascent chiasmata. Scale bars = 10 µm for panels A–L; 1 µm for panels M–P.

The SYCP1 staining associated with centromeres and nascent chiasmata during diplonema could reflect the retention of fragments of normal SC, or modified structures peculiar to these sites. To begin to distinguish these possibilities, we examined the localization of three SC central element proteins, SYCE1, SYCE2 and TEX12, in addition to SYCP1 [59]–[61]. As shown in Figure 2, all four central element components localize to sites of paired centromeres and chiasmata during diplonema, consistent with the idea that normal SC fragments are retained at these sites.

### SC Remnants Associated with Centromeres and Chiasmata Are Distinct and Independent

**Figure 3:**
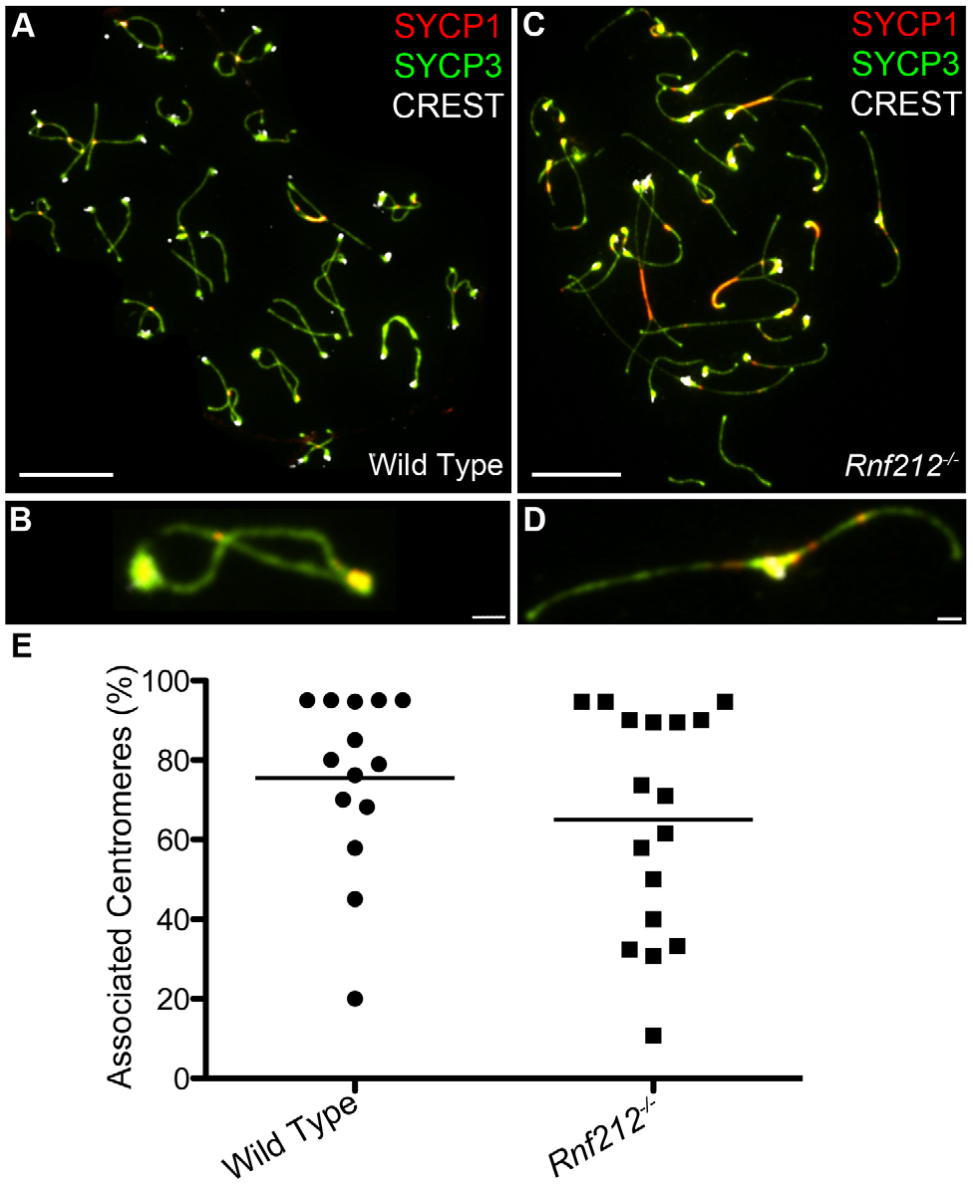
Centromere association in the absence of crossing-over. Representative diplotene-stage spermatocytes from wild-type (A and B) and *Rnf212^−/−^* (C and D) mice, immunostained for SYCP3 (green), SYCP1 (red) and CREST (white). Selected homolog pairs are magnified in panels B and D. Note the absence of chiasmata in D, with homologs remaining associated solely via their centromeres. (E) Levels of associated centromeres in diplotene spermatocytes from wild-type and *Rnf212^−/−^* mice. The two distributions are not statistically different (*P* = 0.35, Mann-Whitney test). Scale bars = 10 µm for panels A and C; 1 µm for B and D.

Previous studies have correlated non-centromeric SC fragments at diplonema with crossover sites (identified as silver-staining recombination nodules [6], [50], [52], [62]) but, to our knowledge, dependency of these structures on crossing-over has not been directly demonstrated. On the other hand, we predict that persistent centromere-associated SC fragments should occur independently of crossing-over. These inferences were tested by analyzing *Rnf212^−/−^* mutant mice, which have normal homolog synapsis, but show a ≥95% reduction of crossing-over (A.R., H.Q., J.K.C. and N.H., unpublished data) (Figure 3). As diplonema progresses and desynapsis ensues in *Rnf212^−/−^* spermatocytes, chromosome arms completely dissociate, but SYCP1-associated centromeres frequently remain connected (Figure 3C–3E). Persistent interstitial SC fragments were not observed in *Rnf212^−/−^* nuclei, indicating dependence on crossing over.

### SC Remnants Are Fragments of Tripartite Synaptonemal Complex

**Figure 4:**
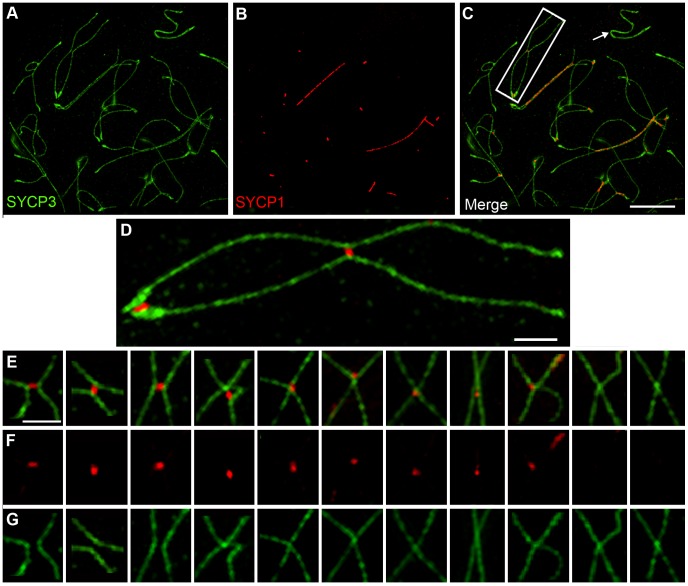
Axis remodeling revealed by structured illumination microscopy of diplotene-stage spermatocytes. All panels show chromosomes from diplotene-stage nuclei immunolabled for SYCP3 (green) and SYCP1 (red). (A–C) A representative diplotene-stage nucleus. The arrow highlights the X-Y chromosome pair. The chromosome highlighted by the white box is magnified in (D). Note the two foci of SYCP1-staining SC central-element localized between the SCYP3-staining homolog axes. Terminal accumulation of SYCP3 indicates the position of the centromeres. (E–G) Selected examples of nascent chiasmata showing various patterns of axis fusion and associated SC central element. Panels in E show the SYCP3 and SYCP1 channels merged; F shows the SYCP1 channel only; G shows the SYCP3 channel only. Scale bars = 5 µm for panels A–C; 1 µm for D–G.

During diplonema, it is not unusual to detect foci of central element components that remain associated with separated homolog axes (e.g. Figure 2). Thus, the association of central element components with centromeres and crossover sites might not represent true tripartite SC, but merely SC remnants associated with only one homolog axis. These two possibilities were discriminated using structured illumination microscopy (SIM), which has sufficient resolving power to distinguish the two SYCP3-stained SC lateral elements from the SYCP1-stained central element (Figure 4) [63], [64]. SIM imaging reveals that centromere and crossover associated SYCP1 is sandwiched between the two homolog axes, as expected for true tripartite SC (Figure 4A–4D).

### Crossover-Associated SC Fragments Precede Axis Remodeling at Chiasmata

SIM analysis of late diplotene nuclei indicates that crossover-associated SC fragments can be very short, comprising on average only 0.24 µm of SYCP1 (Figure 4), less than 3% of the length of an average late pachytene SC (8.6 µm). Moreover, in 41% of these structures (29/71 from 6 nuclei), the SYCP3-staining homolog axes remain clearly separate implying that they have yet to be exchanged to form chiasmata (Figure 4E–4G). Notably, in mouse, DNA exchange to form crossovers has been shown occur during pachynema [65], [66]. Thus, DNA crossing-over and axis-remodeling appear to be temporally distinct aspects of chiasma formation.

Intriguingly, the 31% of nascent chiasmata sites in which SYCP3 axes converge and begin to fuse (22/71) are associated with smaller and less intense SYCP1 foci, which are typically localized to one side of the presumed axis-exchange point (Figure 4E–4G). Finally, 28% of nascent chiasmata (20/71) comprised SYCP3 fusion-points without associated SYCP1.

### SC Central Element Prevents Unregulated Fusion of Homolog Axes at Recombination Sites and Chromosome Ends

**Figure 5:**
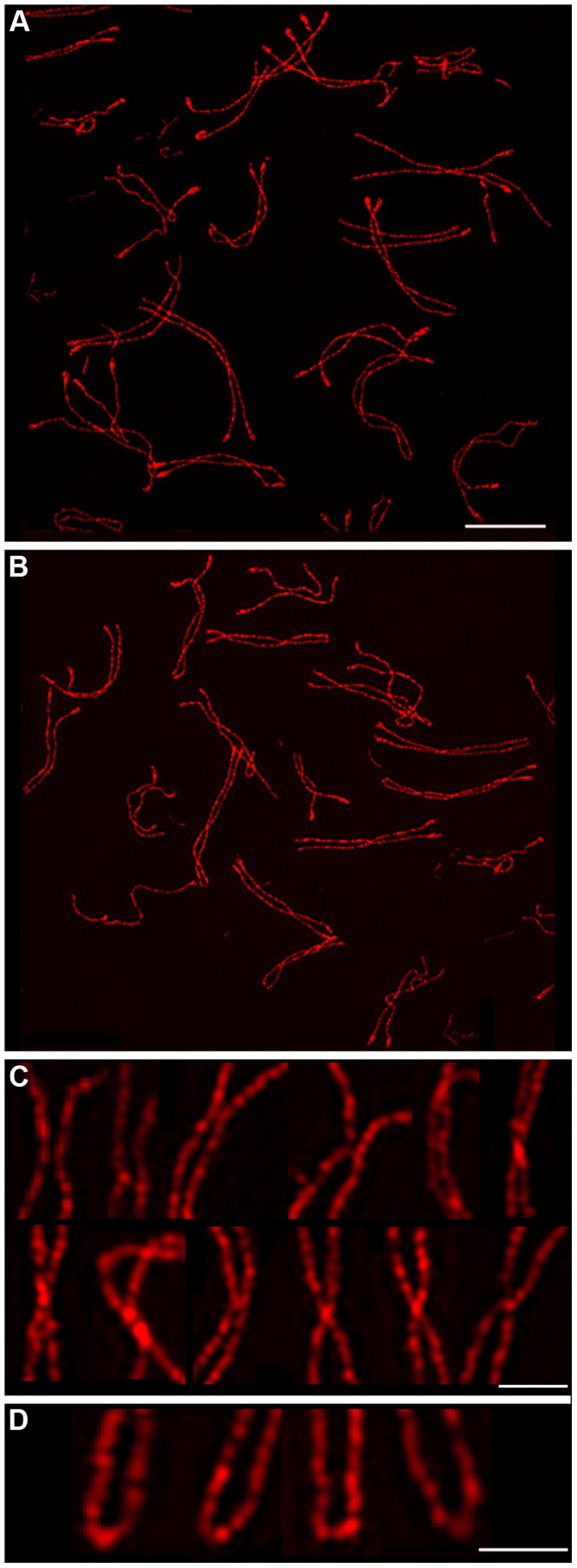
Chiasma-like structures and terminal fusions in the absence of synapsis and crossing-over. (A and B) Representative diplotene-like nuclei from the *Sycp1^−/−^* knock-out immunostained for SYCP3. (C) Gallery of selected examples of axial association sites showing a variety of axis morphology: clearly separated, touching, converging and fused. (D) Fusion of non-centromeric termini into terminal loops. Scale bars = 5 µm for panels A and B; 1 µm for C and D.

The analysis above suggests that SC fragments are retained at crossover sites to regulate the exchange of homolog axes. To further explore this idea, we performed SIM analysis of diplotene-like nuclei from the *Sycp1^−/−^* mouse, which fails to form SC central element (Figure 5A, 5B). Although synapsis fails in *Sycp1^−/−^* meiocytes, the early steps of recombination occur normally, homologs pair and axes closely associate at sites of recombination (so called, “axial associations”) [17]. In nuclei with late-stage chromosome morphology, previously defined to be in diplonema [17], axial associations appeared as a mixture of separate and conjoined/fused SYCP3 axes (Figure 5C), similar to the nascent chiasmata observed in wild-type diplotene nuclei. However, chiasma-like structures in *Sycp1^−/−^* nuclei are distinct from those in wild type. First, they are more numerous, averaging 1.8 (±1.1 SD, n = 89) per homolog pair compared to 0.96 (±0.63 SD, n = 74) in wild type (*P*<0.0001, Mann-Whitney test). Second, 87.3% (137/157) of chiasma-like structures in *Sycp1^−/−^* cells have conjoined or fused axes compared to 59.1% in wild type (42/71; *P*<0.0002, z-test). This observation suggests that nascent chiasma sites with separate SYCP3 axes are stabilized by SC central elements. Moreover, given that crossovers are almost completely abolished by *Sycp1* mutation [17] (as shown by the absence of both crossover-specific MLH1 foci and chiasmata), the observed chiasma-like structures are forming independently of interhomolog crossing over.

In addition, in *Scyp1^−/−^* diplotene cells, we repeatedly observed chromosomes in which the non-centromeric ends of the homolog axes had fused to form contiguous terminal loops (Figure 5D); 15.2% of homolog pairs (14/89) had such structures. Analogous terminal loops were never observed in wild-type diplotene-stage nuclei imaged by SIM. Both the chiasma-like structures and terminal fusions detected in *Sycp1^−/−^* spermatocytes suggest a tendency for unregulated interactions between homolog axes in the absence of SC central element.

### Features of Diplotene Centromeres Revealed by Structured Illumination Microscopy

**Figure 6:**
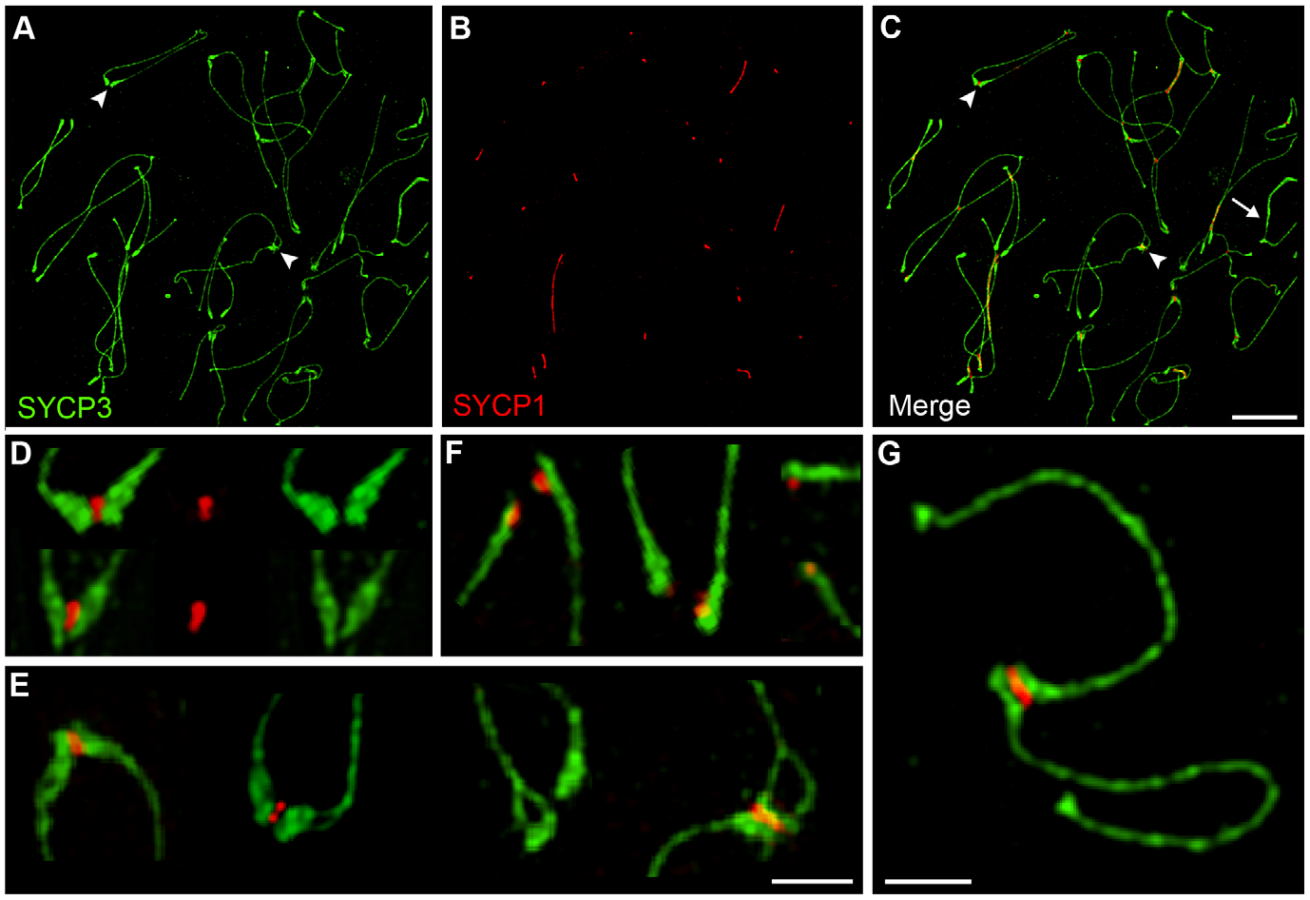
Centromere-associated SC fragments during diplonema. (A–C) A representative diplotene-stage nucleus immunostained for SYCP3 (green) and SYCP1 (red). Arrowheads indicate paddle-like terminal structures with and without axis splitting. The arrow in C highlights the X-Y chromosome pair. (D) Selected examples of associated centromeres highlighting individual SYCP3-staining homolog axes connected by SYCP1-staining SC central element. (E) Selected examples of associated centromeres showing varying degrees of axis splitting. (F) Selected examples of separated centromeres showing retention of SYCP1. (G) Example of an ostensibly achiasmate homolog pair connected solely by SC-associated centromeres. Scale bars = 5 µm for panels A–C; 1 µm for D–G.

Several characteristics of SC-associated centromeres in diplonema were also refined by SIM analysis (Figure 6). First, the well-characterized accumulations of SYCP3 at the centromeric termini [47] form paddle-like structures that can be more than three times broader than the homolog axes (Figure 6A and 6C, highlighted by an arrowhead, and 6D). Second, 7% (12/172) of these terminal SYCP3 structures show a dual morphology suggestive of sister-chromatid individualization; in fact, clear examples of associated centromeres with split sister-axes were observed (Figure 6A and 6C, highlighted by an arrowhead, and 6E). Third, dissociated or even widely separated centromeres can retain SYCP1 staining (Figure 6F). This observation raises the possibility that final desynapsis of centromeres may not occur by simple dissociation of central element proteins from the axes, but by separation of central element transverse filaments (comprising SYCP1 and other proteins) that connect homolog axes via a head-to-head configuration of overlapping homodimers [6]. Alternatively, centromeric connections may have become sufficiently weak that they are mechanically disrupted during the spreading procedure. Fourth, we observed several examples of ostensibly achiasmate homologs, without an internal SYCP3 connection, that remain connected by synapsed centromeres (4/74 homolog pairs; Figure 6G). Whether these homologs are truly achiasmate and their ultimate segregation fate remains unclear, but their detection is consistent with our observation that persistent centromere synapsis occurs independently of crossing-over (above). The possibility that persistent centromere synapsis facilitates the segregation of achiasmate chromosomes is also raised by this observation. Finally, the centromeric ends of the X and Y chromosomes do not show the dramatic SYCP3 accumulation and morphological changes seen for autosomes at this stage. Instead, a general accumulation of SYCP3 signal along the lengths of the X-Y pair is observed (Figure 6C; highlight by and arrow).

### Centromere Association during Diplonema Is Mediated by the SC Central Element

**Figure 7:**
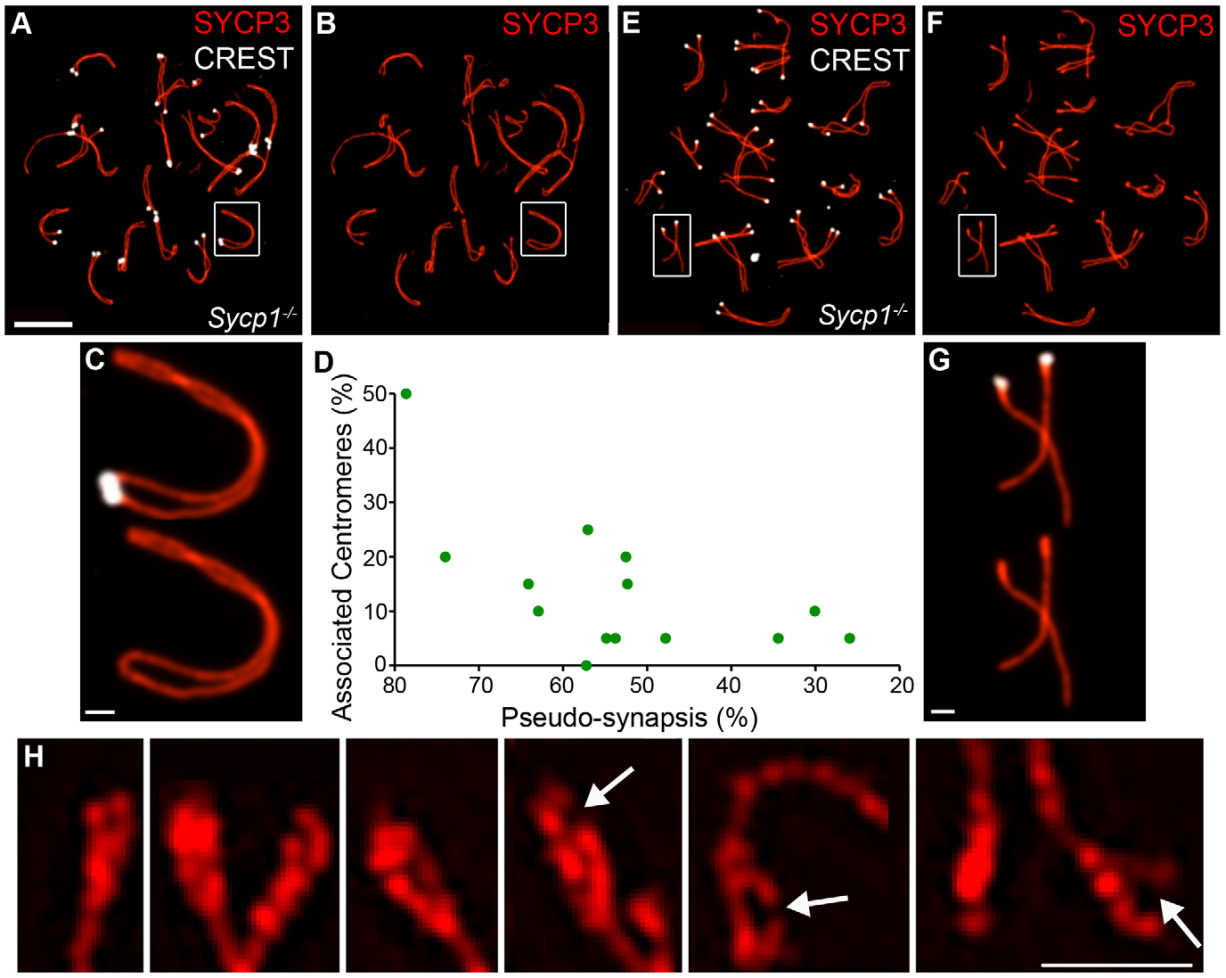
Centromere association and morphology of centromere regions in the absence of synapsis. Spread spermatocyte nuclei from the *Sycp1^−/−^* knock-out immunostained for SYCP3 (red) and CREST (white). (A and B) Pachytene-like nucleus showing extensive coalignment or pseudo-synapsis of homologous chromosomes and a significant fraction of associated centromeres. The chromosome pair highlighted by a white box is magnified in panel (C). Note that the centromeres are associated and the centromeric termini are clearly fused into a terminal loop. (D) Levels of centromere association in pachytene-like *Sycp1^−/−^* nuclei plotted as a function of the level of pseudo-synapsis. (E and F) Representative diplotene-like nucleus in which homologs remain connected at one or more sites but the centromeres are clearly separated. The homologs highlighted by a white box are magnified in panel G. Note the thickened SYCP3 centromeric termini typical of diplotene nuclei. (H) Structured illumination microscopy images of selected centromeric termini from diplotene-like *Sycp1^−/−^* nuclei showing duality, splitting and fracturing (fractured and split axes are highlighted by arrows). Scale bars = 10 µm for panels A, B, E and F; 1 µm for C, G and H.

The analysis above implies that both the association of centromeres in pachynema and their continued connection during diplonema are mediated by SC central element. To test these inferences, we examined centromere association in spermatocytes from the synapsis-defective *Sycp1^−/−^* mutant (Figure 7). For individual pachytene-like *Sycp1^−/−^* spermatocyte nuclei, we determined the frequency of associated centromeres as well as the extent of homolog coalignment or “pseudo-synapsis”, which was defined as the fraction of homolog axes that were separated by ≤0.8 µm (Figure 7A–7D; 0.8 µm was determined to be the maximum distance measured between regions of coaligned axes in *Sycp1^−/−^* nuclei). This analysis indicates that centromere association is not absolutely dependent on the SC central element (Figure 7D). In fact, we observed several examples in which centromeric ends of homologous SYCP3 axes appear fused with one another to form a contiguous loop, even though adjacent regions are clearly separated (e.g. Figure 7B and 7C). These terminal fusions are distinct from those observed in diplotene-like nuclei, which involve the non-centromeric chromosome ends (above and Figure 5D). Overall, however, centromere pairing in *Sycp1^−/−^* spermatocytes never reaches wild-type levels and centromere regions remain the last to pair. Even in nuclei with >70% pseudo-synapsis, ≤50% centromere pairing is observed.

In contrast to the pachytene-like nuclei analyzed above, centromeres are not associated in diplotene-like *Sycp1^−/−^* nuclei even though homologs remain stably tethered by one or more axial association (Figure 7E–7G; 0/89 homolog pairs analyzed by SIM had associated centromeres). Thus, persistent centromere association during diplonema is SYCP1 dependent.

### SIM Analysis of Centromeric SYCP3 Structures from *Sycp1* Mutant Spermatocytes

A number of possible functions can be imagined for centromeric SC fragments during diplonema. For example, continued synapsis of centromeres could resist or maybe even promote the splitting of sister-axes that is detected at this stage (described above; Figure 6E); or it could promote the reorganization of centromere regions, such as the accumulation of SYCP3, modification of cohesion and assembly of kinetochore components; or persistent centromere synapsis could indirectly facilitate homolog biorientation, for example by helping establish connections between centromeric heterochromatin similar to those described in *Drosophila* [67].

To begin to explore these possibilities, SYCP3-stained diplotene-like nuclei from *Sycp1^−/−^* spermatocytes were imaged by SIM (Figure 7H; also see Figure 5). Thickening of the SYCP3-stained centromeric termini was still clearly apparent in *Sycp1^−/−^* mutants (Figure 7H). However, duality and splitting, indicative of sister-chromatid individualization, was exaggerated in *Sycp1^−/−^* cells (Figure 7H), being observed at 19.2% (34/177) of centromeric termini compared to 7% (12/172) of wild-type ends (*P* = 0.0007, z-test). Moreover, *Sycp1^−/−^* centromeric termini had a more fragile and fractured appearance (Figure 7H, arrows highlight gaps or fractures); 13.6% (24/177) of centromeric ends had clear gaps or breaks, a morphology that was never observed in wild-type cells. Thus, diplotene-stage centromeric SYCP3 structures appear to be stabilized by continued synapsis.

### Evidence for Persistent Centromere Linkages after SYCP1 Dissociation

SYCP1 staining is lost and homologous centromeres desynapse in late diplonema (above). Therefore, unlike Zip1-mediated coupling in budding yeast, persistent centromeres synapsis seems unlikely to play a direct role in promoting the stable biorientation of homologs on the meiosis I spindle, which doesn't assemble until diakinesis when the nuclear membrane breaks down.

**Figure 8:**
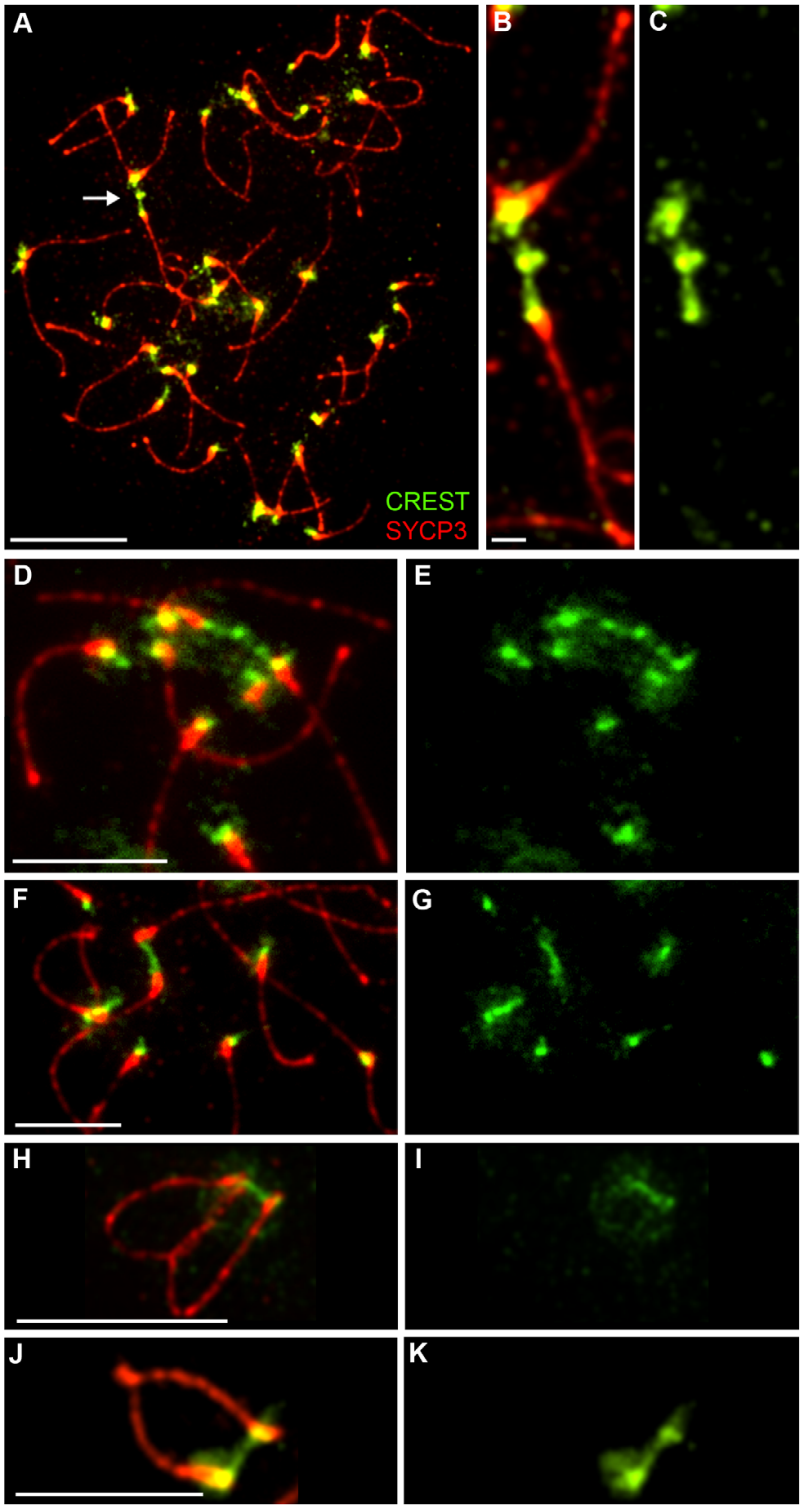
Identification of post-synapsis inter-centromeric CREST-staining bridges. (A) Selected image of a late diplotene/early diakinesis spermatocyte from an *Rnf212^−/−^* mouse, immunostained for SYCP3 (red) and CREST (green). The arrow in panel A highlights the chromosomes magnified in panels B and C. Additional examples of CREST-staining bridges are shown in D–G. (H–K) Examples of CREST-staining bridges from wild-type spermatocytes. Scale bars = 10 µm for panel A; 1 µm for B and C; 5 µm for D–K.

Although homologous centromeres desynapse during late diplonema, we noted that they often appear closely associated and oriented towards one another, even in the absence of chiasmata (in *Rnf212^−/−^* spermatocytes). Moreover, we routinely detected inter-centromeric CREST-staining structures at this stage (Figure 8) giving the impression of interconnecting chromatin bridges. However, distinct DAPI-staining bridges cannot be discerned at this stage because the chromatin is very diffuse (not shown).

**Figure 9:**
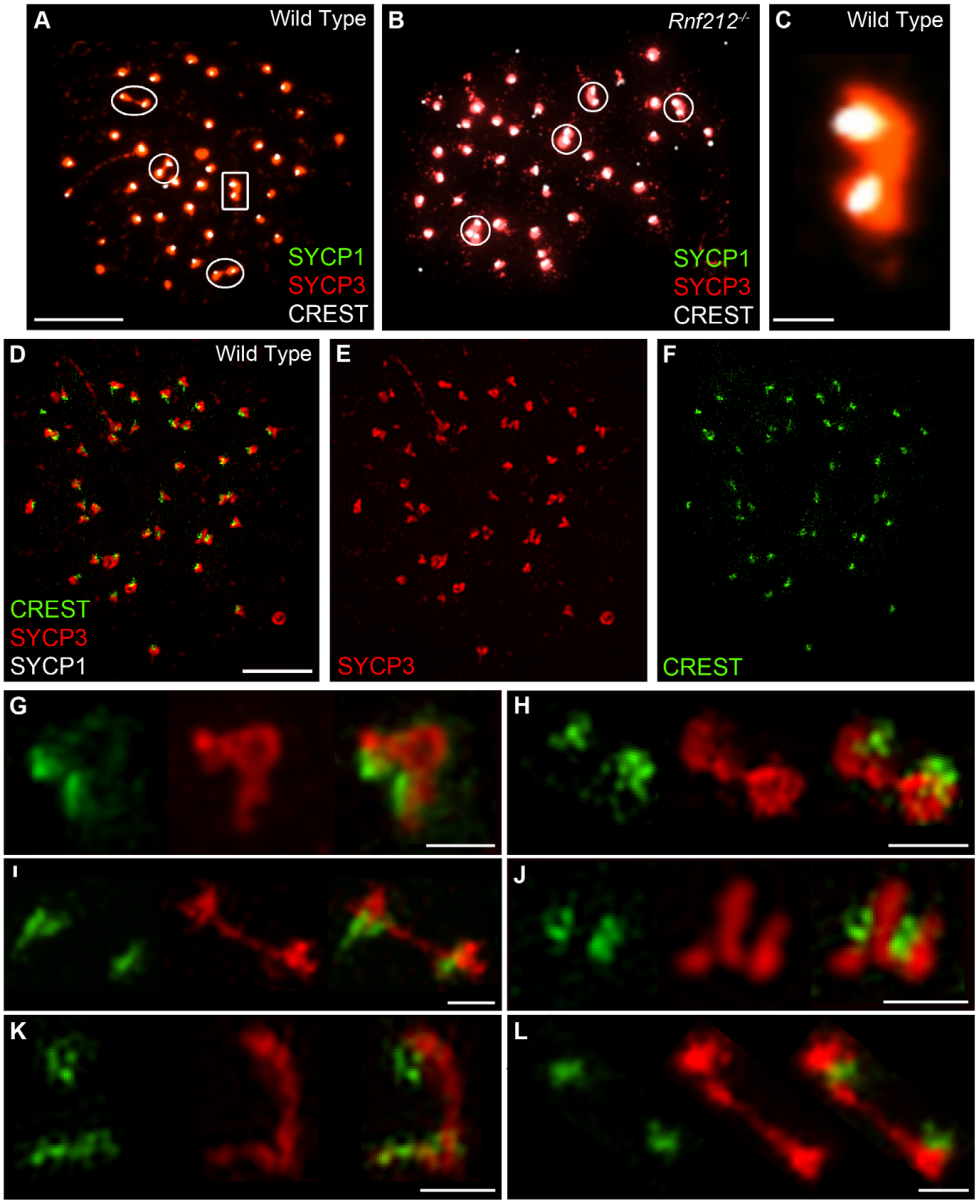
Identification of inter-centromeric SYCP3-staining bridges in diakinesis/metaphase I spermatocytes. (A–C) Selected diakinesis/metaphase-I stage spermatoytes from wild-type and *Rnf212^−/−^* mice, immunostained for SYCP3 (red), SYCP1 (green), and CREST (white). Circles highlight pairs of CREST foci associated with interconnected bi-lobed SYCP3 structures. The white rectangle in A indicates the SYCP3 structure magnified in panel C. Note the absence of SYCP1 staining at this stage. (D–L) Selected SIM images of diakinesis/metaphase-I stage spermatocytes from wild-type mice, immunostained for SYCP3 (red), SYCP1 (white) and CREST (green) (note that only SYCP3 and CREST channels were imaged by SIM; SYCP1 was imaged in the same microscope by conventional epifluorescence to confirm the absence of SYCP1 staining at this stage). Panels G–L show a gallery of selected pairs of CREST structures interconnected by contiguous SYCP3-staining structures. Note the duality of most CREST-staining structures, consistent with the individualization of sister-kinetochores. Scale bars = 10 µm for panels A and B; 1 µm for C; 5 µm for D–F; 1 µm for G–L.

As described previously, axial SYCP3 mostly disappears from diakinesis/metaphase-I chromosome axes to leave only faint interchromatid foci that define the chiasmata (e.g. Figure 1N) [47]. In contrast, centromeric SYCP3 becomes more abundant and remains closely associated with CREST-staining kinetochores [47], [68], [69]. Intriguingly, in diakinesis/metaphase I nuclei, we regularly detected closely apposed CREST signals associated with apparently contiguous, bi-lobed SYCP3 structures, or structures that are connected by thin SYCP3-staining strands (Figure 9A–9C). The close apposition of the centromeres in these structures could, in theory, be caused by proximal chiasmata (although crossover-specific MLH1 foci are rarely found close to centromeres). However, analysis of crossover-defective *Rnf212^−/−^* mutant nuclei indicates that they arise independently of chiasmata (Figure 9B). On average, around two SYCP3-linked centromere pairs were observed in both wild-type and *Rnf212^−/−^* spermatocytes (1.9±1.8 SD, n = 13 and 2.2±1.4 SD, n = 18).

SIM analysis of diakinesis/metaphase-I nuclei supports the inference that these linked centromere-pairs are associated with contiguous SYCP3-staining structures (Figure 9D–9L). In one example, discontinuous CREST staining also appears to bridge between the two homolog-kinetochores (Figure 9G). Taken together, our observations support the possibility that the centromere regions of homologs can remain interconnected long after they have desynapsed during late diplonema.

## Discussion

### Centromeres Do Not Drive Homolog Synapsis in Mammals

In contrast to budding yeast and *Drosophila,* mouse centromeres appear to be refractory to SC formation and are the last sites to synapse. The same conclusions are drawn by Bisig et al. in the accompanying study [48]. The possibility that this is a general feature of mammalian meiosis is supported by the study of Hassold and colleagues, which showed that centromeres of human spermatocyte chromosomes constitute a barrier to the polymerization of SC [11], [53]. In fact, the early synapsis of budding yeast and *Drosophila* centromeres may be more the exception than the rule, as late pericentric synapsis is typical of many organisms including fungi, plants and mammals [11], [49]–[53].

We conclude that centromeres do not drive SC formation in mammals, but are in fact refractory to synapsis. This is true despite the fact that during zygonema, mouse centromeres cluster into a single large chromocenter, which might have been expected to facilitate centromere synapsis [70]. Indeed, recent studies in *Drosophila* females show that synapsis initiates within the chromocenter, indicating a fundamental difference with mammals [19], [30], [31]. We suggest that mammalian centromeres are synapsed only after SC polymerization switches from the initial homology-dependent, recombination-driven phase to the well-characterized (but poorly understood) homology-indifferent “synaptic adjustment” mode [6], [71].

How and why mammalian centromeres resist synapsis remains unclear. Random (non-homologous) association of centromeric major satellite DNA within chromocenters could oppose forces that attempt to drive homologous pairing and synapsis of centromeres. Also, suppression of recombination initiation close to centromeres could limit not only local SC initiation, but also the extension of SC polymerization from adjacent sites. Another possibility is that chromatin and/or axial structures associated with centromeres are modified in ways that impede early synapsis. Notably, Roig et al. [51] showed that centromere synapsis is unusually dependent on the AAA+ ATPase, TRIP13, which facilitates the removal of HORMA-domain proteins from synapsing homolog axes [72].

SC initiation sites have been correlated with crossing-over in a number of organisms. However, it remains unclear whether crossover-designation triggers SC formation, or SC initiation sites trigger crossing-over [2], [6], [11], [73]. Under the latter scenario, absence of SC initiation between centromeres could function to suppress crossing-over within the multi-megabase, repetitive DNA elements that constitute mammalian centromeres [74]. This in turn will minimize the risk of chromosome rearrangements that could result from non-allelic centromere exchanges. Suppression of allelic crossing-over near centromeres will also help minimize the incidence of nondisjunction, which has been associated with such events in yeast, flies and humans [27], [75]. By contrast, the tiny size of budding yeast centromeres (125 bp) makes them highly unlikely to engage in non-allelic crossing-over. We also note that although *Drosophila* centromeres serve as SC initiation sites [30], [31], they may be protected from meiotic instability by the fact that SC initiation sites do not correlate with crossing-over in this organism. Moreover, homolog pairing and synapsis are not driven by recombination in *Drosophila*.

### Crossover-Dependent SC Fragments May Stabilize Nascent Chiasmata and May Coordinate Axis Exchange and Bivalent Maturation

Our analysis implies that DNA crossing-over and axis remodeling (at least with respect to SYCP3) are temporally distinct aspects of chiasma formation. Analysis of *Sycp1^−/−^* spermatocytes suggests that this temporal separation may be mediated by the SC central element. Furthermore, the chiasma-like structures and terminal fusions observed in *Sycp1^−/−^* mutants suggest a novel role for SC central element in preventing the unregulated fusion and exchange of homolog axes.

After diplonema, chromatin condenses and sister-chromatids individualize to become located on opposite sides of their cohesin axis. As bivalents further condense, chromatids also bend sharply at sites of crossing-over [14]. The requisite local flexibility appears to be reflected by two morphological features of crossover sites: relaxation of sister-chromatid cohesion and reduced chromatin condensation [6]. We suggest that SC fragments could help implement these features by triggering local loss of cohesin and/or differential loading of condensin (the loading of which may be coupled to SC disassembly [76]).

A role for crossovers in bivalent remodeling has been clearly demonstrated in *C. elegans*. In this organism, crossover sites trigger asymmetric loss of SC components and, subsequently, cohesion from bivalent arms [77], [78]. This global remodeling of bivalents may be peculiar to organisms with holocentric chromosomes. In organisms with conventional centromeres, in which all arm cohesion is lost at anaphase I, we suggest that crossovers only trigger local changes in cohesion and chromatin condensation, as described above.

### A Role for Diplotene SC Fragments in Centromere Function?

SIM analysis has revealed a tendency for local separation of sister-chromatid axes at synpased centromeres during diplonema. Kleckner et al. have proposed that cycles or chromatin expansion and contraction drive such transient individualization of sister-chromatids in order to facilitate chromosome remodeling and installation of components required for subsequent stages [79]. The enhanced splitting of centromeric SYCP3 structures seen in *Sycp1^−/−^* mutants supports the idea that SC fragments retained at diplonema are part of a supporting framework that constrains and targets local expansion to help coordinate remodeling at centromere regions.

The chromosomal passenger complex (CPC) regulates and orchestrates several key processes during chromosome segregation and cell division. These include sister-chromatid cohesion, kinetochore-microtubule attachments, spindle stability and cell division [80]. In addition, during meiosis the CPC regulates the timing of SC disassembly [76], [81], [82]. Cytological analyses of mouse spermatocytes have shown that CPC components, INCENP and Aurora-B, relocalize from centromeric heterochromatin to the inner centromere domain during diplonema, i.e. concurrent with the retention of SC at centromeres [83], [84]. In addition, INCENP associates with the SC central element [84]. These observations raise the intriguing possibility that centromere-associated SC fragments in diplonema facilitate CPC relocalization and initial stages of kinetochore maturation.

### What Are the Signals for Local SC Retention?

Centromere pairing in budding yeast requires PP4-dependent dephosphorylation of the SC component, Zip1 (which is phosphorylated in response to DSB formation; [23]). In addition, the budding yeast SC central element component, Zip1, can bind SUMO, which is a prominent modification at centromeric heterochromatin and kinetochores [56], [85]. In *Drosophila* females, the CPC stabilizes SCs presumably by antagonizing kinases that promote SC disassembly (see above [76], [81]). Thus, the high concentration of CPC at spermatocyte centromeres could promote local resistance to SC disassembly. How crossovers signal local retention of SC remains mysterious. In rat, the crossover marker, CDK2, remains at crossover sites until diplonema and could signal SC retention [86]. However, in male mice, CDK2 does not obviously persist at crossover sites beyond pachynema [87].

### Post-Synapsis Bridges Suggest Tethering of Homologous Centromeres

Persistent association of centromeres throughout diplonema appears to be a conserved feature of meiosis in many organisms, including budding yeast, *Drosophila* and mouse ([4], [21], [30], [32], [50], [52], [62] and this study). In budding yeast, late centromere coupling promotes the correct, bipolar (syntelic) attachment of chiasmate bivalents to the spindle and thereby limits engagement of the spindle assembly checkpoint to correct misalignments. Coupling also serves as a backup mechanism for the disjunction of occasional achiasmate chromosomes [21], [32], [33]. The role of late centromere synapsis in *Drosophila* remains unclear, but association of centromeric heterochromatin is important for achiasmate segregation in this organism [88]–[90].

It seems unlikely that the persistent centromere synapsis observed in mouse is directly analogous to centromere coupling in budding yeast. Notably, centromere synapsis does not persist beyond diplonema so that a direct role in homolog biorientation and achiasmate disjunction is not envisioned. However, coupling could theoretically function indirectly in these processes by promoting centromere association, orientation and/or the organization of kinetochores prior to nuclear envelope breakdown and spindle assembly.

The inter-centromeric CREST-staining bridges we detect in late-diplotene/early diakinesis cells are reminiscent of the heterochromatin threads that connect achiasmate (and perhaps chiasmate) chromosomes during meiosis in *Drosophila* females [67]. These structures are proposed to facilitate the congression of achiasmate chromosomes during prometaphase and promote their disjunction at anaphase I. The achiasmate X-Y disjunction systems found in some mammals appear to use specialized structures, derived from SC components, to tether the X and Y chromosomes [35]–[37]. The inter-centromeric CREST bridges and SYCP3 structures that we detect in diakinesis/metaphase-I spermatocytes might reflect the existence of related processes in mouse that can favor the biorientation of homologous centromeres and/or facilitate the disjunction of chromosomes that have failed to crossover.

## Materials and Methods

### Ethics Statement

All experiments conformed to relevant regulatory standards and were approved by the U.C Davis Institutional Animal Care and Use Committee.

### Mice

All mice were congenic with the C57BL/6J background. The *Sycp1* and *Spo11* knock-out lines were previously described [17], [91]. Generation of the *Rnf212* knock-out line will be described elsewhere (Reynolds et al., submitted). PCR genotyping of *Rnf212* mice was performed using primers exon forward (5′-CGCTGGAATGAACGCAGGCGC-3′), exon reverse (5′-CAGGGGAGTGAAGCCACGGTC-3′), pH530 (5′-TCCATGGGCTTAAACCAGTGC-3′), and VM3 (5′-GCGCATGCTCCAGACTGCCTTG-3′). Primers, exon forward and exon reverse, generate a 290-bp fragment diagnostic of the *Rnf212* wild-type allele; pH530 and VM3 detect the *Rnf212* mutant allele as a 383-bp fragment. PCR conditions were 30 seconds at 94°C, 30 seconds at 60°C, and 1 minute at 72°C for 30 cycles.

### Cytology

Testes were removed from 2–4 month old mice and processed for surface spreading as described [92]. Immunofluorescence staining was performed as described [93] using the following primary antibodies overnight at room temperature (dilutions in parentheses): rabbit anti-SYCP3 (sc-33195 Santa Cruz, 1∶300); mouse anti-SYCP3 (sc-74568 Santa Cruz, 1∶200); mouse anti-rat SYCP1 monoclonal antibody [94] (1∶400); CREST antiserum (generously provided by Shelby White, ARUP Laboratories; 1∶10000); mouse monoclonal anti-γH2AX (05-636 Millipore, 1∶500), rabbit anti-mouse RAD21L (a generously gift of K. Ishiguro and Y. Watanabe, University of Tokyo [45] (1∶200); guinea pig anti-SYCE1 (1∶2000), guinea pig anti-SYCE2 (1∶400) and guinea pig anti-TEX12 (1∶200) [95], [96]. Slides were subsequently incubated with the following goat secondary antibodies for 1 hour at 37°C: anti-rabbit 488 (A11070 Molecular Probes, diluted 1∶10000), anti-rabbit 568 (A11036 Molecular Probes, diluted 1∶2000), anti-human 488 (A11013 Molecular Probes, 1∶2000), anti-mouse 594 (A11020 Molecular Probes, 1∶10000), anti-human DyLight 649 (109-495-088 Jackson Labs, 1∶200), and anti-guinea pig fluorescein isothiocyanate (106-096-006 FITC, Jackson Labs, 1∶200). Coverslips were mounted with ProLong Gold antifade reagent (Molecular Probes).

### Imaging

Immunolabeled chromosome spreads were imaged using a Zeiss AxioPlan II microscope with 63× Plan Apochromat 1.4 objective and EXFO X-Cite metal halide light source. Images were captured by a Hamamatsu ORCA-ER CCD camera. Image processing and measurements were performed using Volocity (Perkin Elmer) and Photoshop (Adobe) software packages. Any pair of CREST foci that was ≤0.6 µm apart was classified as associated; convergent SYCP1 staining defined synapsed centromeres. To account for overlapping CREST foci, total numbers of CREST foci were counted for all nuclei. In nearly all cases, overlapping pairs of CREST foci could be discerned as larger, more intense, bi-lobed staining structures. Only nuclei for which all centromeres could be accounted for were used to determine levels of centromere association/synapsis. SIM analysis was performed using a Nikon N-SIM super-resolution microscope system and NIS-Elements 2 image processing software.

## Supporting Information

Figure S1 Synapsis does not initiate at centromeres in the absence of recombination. Spermatocytes from *Spo11^−/−^* knock-outs show a general defect in homolog pairing and synapsis, but a fraction of spermatocytes show significant levels of SC formation, which frequently involves non-homologous chromosomes ([91], [97]). We analyzed zygotene-like *Spo11^−/−^* nuclei to determine whether initial stretches of SC were associated with centromeres (A, B, E and F). For 69 SC stretches (from 10 nuclei), only 6 included the centromeres. Therefore, synapsis does not preferentially initiate between centromeres in the absence of recombination. Levels of centromere association were also determined and plotted as a function of the synapsis level of individual nuclei (C, D and F). Consistent with our analysis of wild-type spermatocytes, high levels of centromere association were only observed in nuclei with high levels of synapsis. This observation supports the inference that polymerization of SC is the major driver of centromere association during meiotic prophase in mouse. (A–D) Representative spermatocyte nuclei from a *Spo11^−/−^* knock out immunolabled for SYCP3 (green), SYCP1 (red), and CREST (white). (E) Magnification of the chromosome indicated by an arrow in A. Synapsis appears to have nucleated between the non-centromeric terminus of a short chromosome and an internal region of a long chromosome. (F) Levels of centromere association as a function of synapsis level in *Spo11^−/−^* spermatocytes. Scale bars = 10 µm for panels A–D; 1 µm for E.
(TIF)

Figure S2 Synapsis does not initiate at centromeres in the absence of mammalian Zip3 ortholog, Rnf212. Analysis of initial SC stretches in zygotene-stage *Rnf212^−/−^* spermatocytes shows that SC formation does not initiate between centromeres (A and B). Only 1 out of 158 SYCP1 stretches was associated with a CREST signal (11 nuclei analyzed). Moreover, centromeres remain among the last regions to synapse (FC–F). Representative early- (A,B) and late-zygotene (C,D) stage spermatocyte nuclei from a *Rnf212^−/−^* knock out immunolabled for SYCP3 (green), SYCP1 (red), and CREST (white). (E and F) Magnification of the chromosome indicated by an arrow in C, highlighting the late synapsis of centromeres. Scale bars = 10 µm for panels A–D; 1 µm for E and F.
(TIF)
