# Supplementary figures and images for: Interplay between Synaptonemal Complex, Homologous Recombination, and Centromeres during Mammalian Meiosis
Source: PLoS Genet. 2012 Jun 28;8(6):e1002790. doi: 10.1371/journal.pgen.1002790 (PMC3386176; doi:10.1371/journal.pgen.1002790)

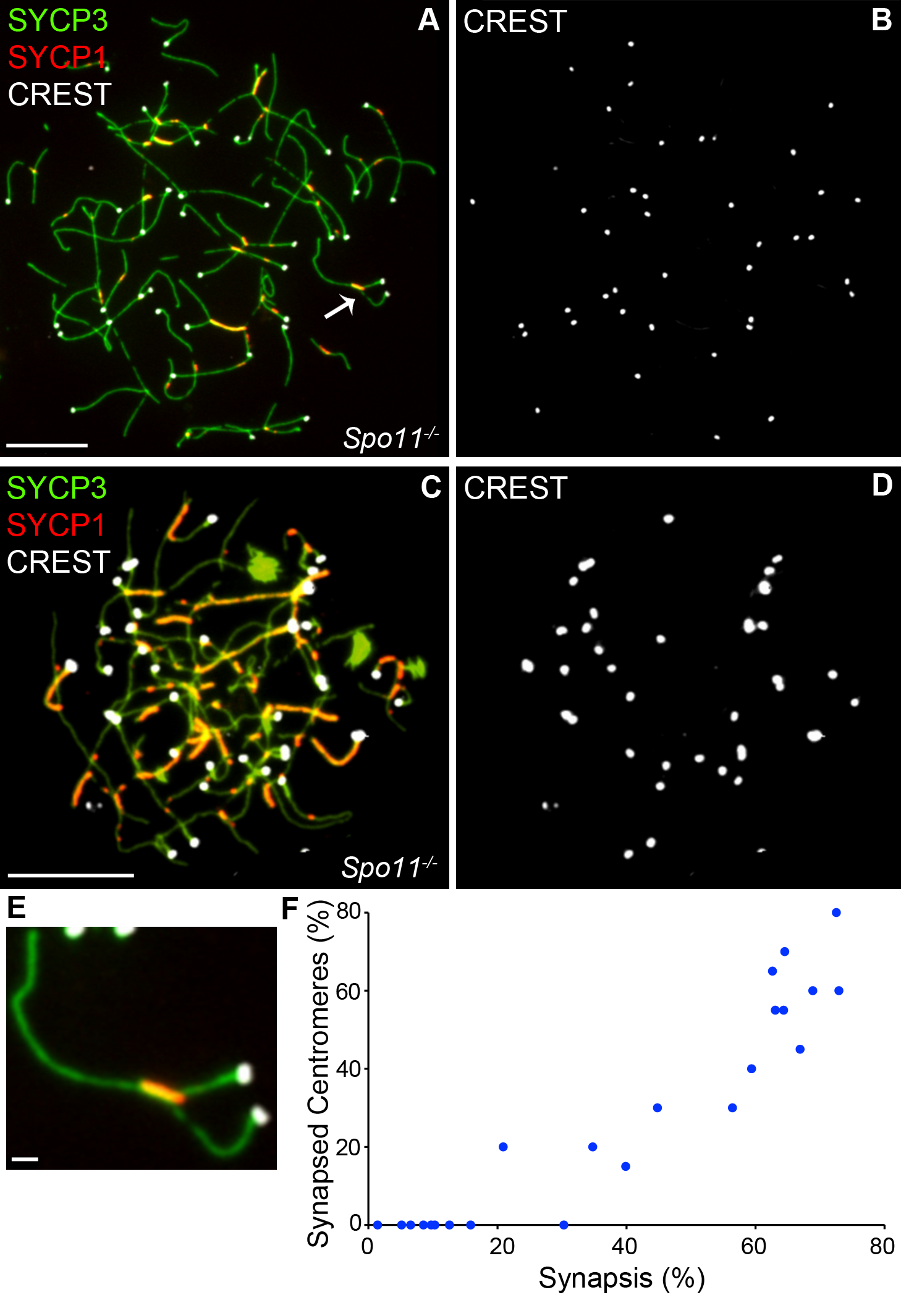

Supplement: Figure S1 — Synapsis does not initiate at centromeres in the absence of recombination. Spermatocytes from Spo11−/− knock-outs show a general defect in homolog pairing and synapsis, but a fraction of spermatocytes show significant levels of SC formation, which frequently involves non-homologous chromosomes ([91], [97]). We analyzed zygotene-like Spo11−/− nuclei to determine whether initial stretches of SC were associated with centromeres (A, B, E and F). For 69 SC stretches (from 10 nuclei), only 6 included the centromeres. Therefore, synapsis does not preferentially initiate between centromeres in the absence of recombination. Levels of centromere association were also determined and plotted as a function of the synapsis level of individual nuclei (C, D and F). Consistent with our analysis of wild-type spermatocytes, high levels of centromere association were only observed in nuclei with high levels of synapsis. This observation supports the inference that polymerization of SC is the major driver of centromere association during meiotic prophase in mouse. (A–D) Representative spermatocyte nuclei from a Spo11−/− knock out immunolabled for SYCP3 (green), SYCP1 (red), and CREST (white). (E) Magnification of the chromosome indicated by an arrow in A. Synapsis appears to have nucleated between the non-centromeric terminus of a short chromosome and an internal region of a long chromosome. (F) Levels of centromere association as a function of synapsis level in Spo11−/− spermatocytes. Scale bars = 10 µm for panels A–D; 1 µm for E. (TIF) [file pgen.1002790.s001.tif]

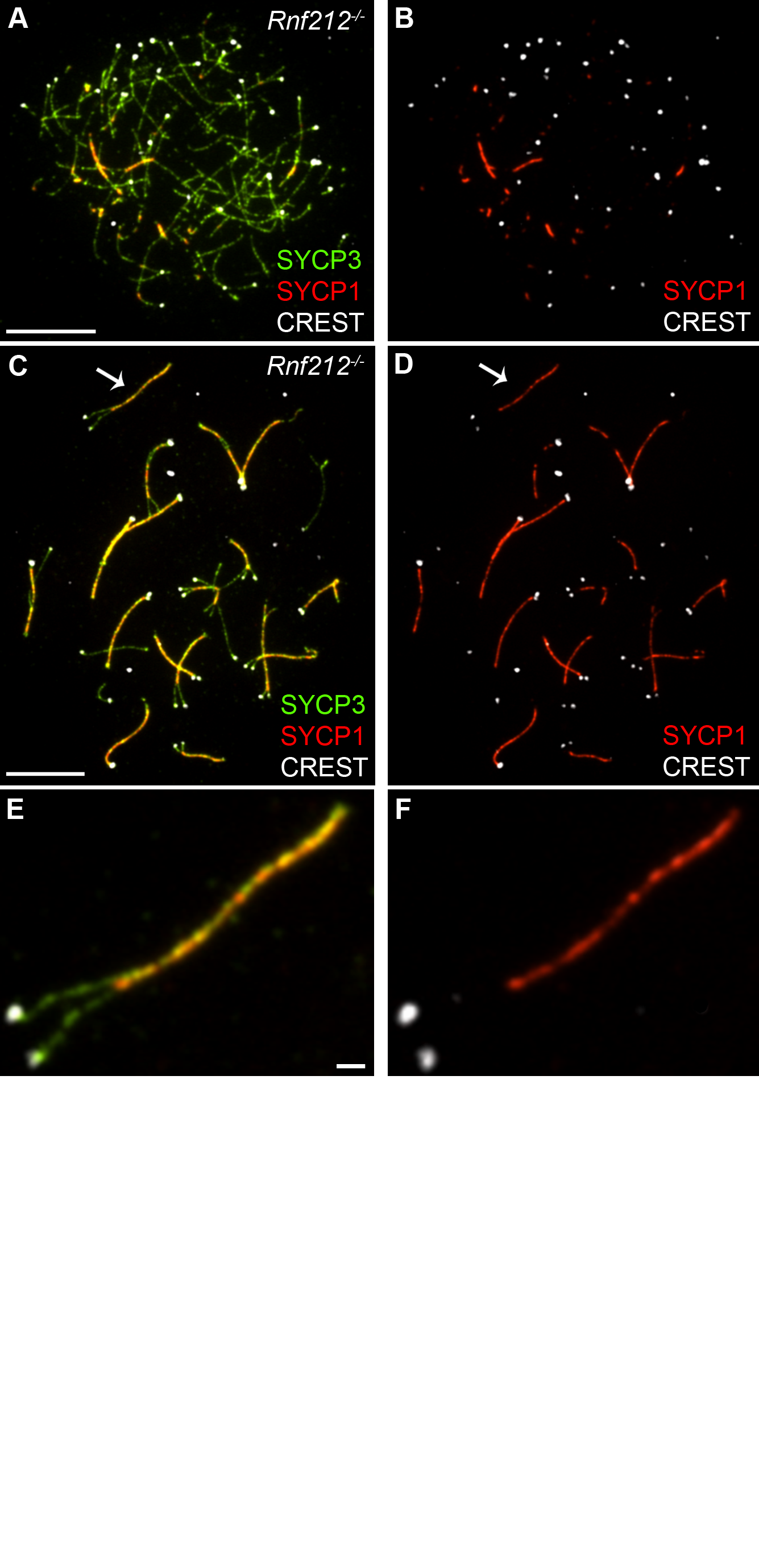

Supplement: Figure S2 — Synapsis does not initiate at centromeres in the absence of mammalian Zip3 ortholog, Rnf212. Analysis of initial SC stretches in zygotene-stage Rnf212−/− spermatocytes shows that SC formation does not initiate between centromeres (A and B). Only 1 out of 158 SYCP1 stretches was associated with a CREST signal (11 nuclei analyzed). Moreover, centromeres remain among the last regions to synapse (FC–F). Representative early- (A,B) and late-zygotene (C,D) stage spermatocyte nuclei from a Rnf212−/− knock out immunolabled for SYCP3 (green), SYCP1 (red), and CREST (white). (E and F) Magnification of the chromosome indicated by an arrow in C, highlighting the late synapsis of centromeres. Scale bars = 10 µm for panels A–D; 1 µm for E and F. (TIF) [file pgen.1002790.s002.tif]
